# The B56γ3 regulatory subunit-containing protein phosphatase 2A outcompetes Akt to regulate p27KIP1 subcellular localization by selectively dephosphorylating phospho-Thr157 of p27KIP1

**DOI:** 10.18632/oncotarget.6609

**Published:** 2015-12-14

**Authors:** Tai-Yu Lai, Chia-Jui Yen, Hung-Wen Tsai, Yu-San Yang, Wei-Fu Hong, Chi-Wu Chiang

**Affiliations:** ^1^ Institute of Basic Medical Sciences, College of Medicine, National Cheng Kung University, Tainan, Taiwan; ^2^ Department of Internal Medicine, College of Medicine, National Cheng Kung University, Tainan, Taiwan; ^3^ Institute of Clinical Medicine, College of Medicine, National Cheng Kung University, Tainan, Taiwan; ^4^ Department of Pathology, College of Medicine, National Cheng Kung University, Tainan, Taiwan; ^5^ Institute of Molecular Medicine, College of Medicine, National Cheng Kung University, Tainan, Taiwan; ^6^ Center for Infectious Disease and Signaling Research, National Cheng Kung University, Tainan, Taiwan

**Keywords:** PP2A, B56γ3, p27, subcellular localization, Akt

## Abstract

The B56γ-containing protein phosphatase 2A (PP2A-B56γ) has been postulated to have tumor suppressive functions. Here, we report regulation of p27KIP1 subcellular localization by PP2A-B56γ3. B56γ3 overexpression enhanced nuclear localization of p27KIP1, whereas knockdown of B56γ3 decreased p27KIP1 nuclear localization. B56γ3 overexpression decreased phosphorylation at Thr157 (phospho-Thr157), whose phosphorylation promotes cytoplasmic localization of p27KIP1, whereas B56γ3 knockdown significantly increased the level of phospho-Thr157. *In vitro*, PP2A-B56γ3 catalyzed dephosphorylation of phospho-Thr157 in a dose-dependent and okadaic acid-sensitive manner. B56γ3 did not increase p27KIP1 nuclear localization by down-regulating the upstream kinase Akt activity and outcompeted a myristoylated constitutively active Akt (Aktca) in regulating Thr157 phosphorylation and subcellular localization of p27KIP1. In addition, results of interaction domain mapping revealed that both the N-terminal and C-terminal domains of p27 and a domain at the C-terminus of B56γ3 are required for interaction between p27 and B56γ3. Furthermore, we demonstrated that p27KIP1 levels are positively correlated with B56γ levels in both non-tumor and tumor parts of a set of human colon tissue specimens. However, positive correlation between nuclear p27KIP1 levels and B56γ levels was found only in the non-tumor parts, but not in tumor parts of these tissues, implicating a dysregulation in PP2A-B56γ3-regulated p27KIP1 nuclear localization in these tumor tissues. Altogether, this study provides a new mechanism by which the PP2A-B56γ3 holoenzyme plays its tumor suppressor role.

## INTRODUCTION

Protein phosphatase 2A (PP2A) is a serine/threonine phosphatase that plays an important role in regulating many aspects of cellular activities [1]. PP2A is a heterotrimeric protein complex, composed of one structural subunit A (PP2A/A), one catalytic subunit C (PP2A/C), and one variable regulatory B subunit (PP2A/B). In mammalian cells, the regulatory B subunits can be grouped into four families named B (also known as B55 or PR55) [1, 2], B' (also known as B56 or PR61) [3, 4], B” (also known as PR72) [5] and B'” (also known as PR93 or PR110) [6]. It has been believed that the substrate specificity and subcellular localization of PP2A is determined by the regulatory B subunits. PP2A has been recognized as a tumor suppressor. Among the diverse PP2A holoenzyme complexes, the PP2A holoenzymes containing the regulatory subunit B56 family have been shown to regulate key molecules involved in tumorigenesis. The B56α-containing PP2A (PP2A-B56α) enhances c-Myc degradation by dephosphorylation of Ser62 of c-Myc [7]. The B56δ-containing PP2A (PP2A-B56δ) acts as a central effector for DNA-damage checkpoint by dephosphorylating cdc25 at Thr138 to control cdc25 association with 14-3-3 [8]. The B56γ-containing PP2A (PP2A-B56γ) holoenzymes have been shown to play an important role in the tumor suppressive activity of PP2A. A truncated B56γ product was found to promote cell motility and metastasis via paxillin phosphorylation in a B16 mouse melanoma cell line [9]. Null or reduced expression of B56γ subunits was found in several human lung cancer cell lines [10] and human malignant melanomas [11], respectively.

B56γ3 overexpression in human lung cancer cell lines partially reversed the tumorigenicity of these cells [10], and knockdown of B56γ can fully transform HEK cell line expressing LT, hTERT, and Ras-V12 [12]. PP2A-B56γ holoenzymes dephosphorylate p53 on Thr55 to stabilize p53, which results in cell cycle arrest, subsequently inhibits cell proliferation and transformation [13]. In addition, a F395C mutation of B56γ in lung cancer specimen was found to be defective in p53 binding [14]. PP2A-B56γ3 holoenzymes regulate p27KIP1 protein levels via downregulation of Thr187 phosphorylation to regulate the G1/S transition of cell cycle progression [15]. In addition, p27KIP1 protein levels regulated by PP2A-B56γ holoenzymes plays a crucial role in cellular transformation mediated by a group of oncogenes, including small t antigen (ST) of SV40 tumor virus [16].

p27KIP1 (hereafter referred to as p27), a KIP family member, is a critical cell cycle regulator that inhibits the cell cycle progression at the G1/S transition [17]. Phosphorylation of p27 at Thr187 targets it for degradation via the SCFSKP2 ubiquitin-proteasome pathway at the G1/S transition [17]. Additionally, phosphorylation of p27 at Ser10 and Thr198 governs its stability in G0 [18–20]. In addition to protein stability, activities of p27 are governed by its subcellular localization, which is regulated by three phosphorylation sites, Ser10, Thr157 and Thr198. Several kinases have been shown to phosphorylate these sites, such as Akt (Ser10, Thr157, and Thr198), human kinase interacting stathmin (hKIS) (Ser10), glucoccoticoid-inducible kinase (SGK) (Thr157) and ribosomal S6 kinase (RSK) (Thr198) [17]. Ser10 phosphorylation induces p27 translocation from the nucleus into the cytoplasm during the G0/G1 transition and early G1 [21]. Similarly, phosphorylation of Thr198 also promotes the cytoplasmic localization of p27 in G0 and early G1 [18, 22]. Thr157 is located within the nuclear localization sequence (NLS) of p27 [22], and phosphorylation of p27 at both Thr157 and Thr198 induces the interaction with 14-3-3 proteins [23], which disrupts the binding of p27 to importin α5 and promotes cytoplasmic localization of p27 [22, 24].

Many cancers show decreased expression of p27 [22], and p27−/− mice show high susceptibility to tumor formation induced by carcinogens or irradiation [25]. On the other hand, rather than displaying reduced expression of p27 like many types of cancer, some types of cancer show p27 mislocalization in the cytoplasm [26]. The cytoplasmic mislocalization of p27 is associated with poor prognosis, higher tumor grade or metastasis in several human malignancies [27]. The stable mislocalized cytoplasmic p27 cooperates with Ras to promote tumor formation [25], and the motility, survival, and tumorigenesis of some tumor cells are also inhibited by reduction of cytosolic p27 [28]. The subcellular localization of p27 regulated by phosphorylation at Ser10, Thr157, or Thr198 by several kinases, such as Akt, hKIS, and RSK, has been well characterized [17]; however, the role of protein phosphatases in this regard has not been elucidated. Here, we demonstrate that the B56γ3-containing PP2A holoenzyme selectively dephosphorylates p27 at Thr157, but not Ser10 or Thr198, to promote nuclear localization of p27. We also demonstrate that regulation of nuclear localization of p27 by dephosphorylation of Thr157 is not through down-regulating Akt activity. Furthermore, we mapped interaction domains of both p27 and B56γ3 and performed IHC staining to investigate the correlation between levels of B56γ and levels of total or nuclear p27 in human colon cancer specimens.

## RESULTS

### B56γ3 increases nuclear localization of p27

**Figure 1:**
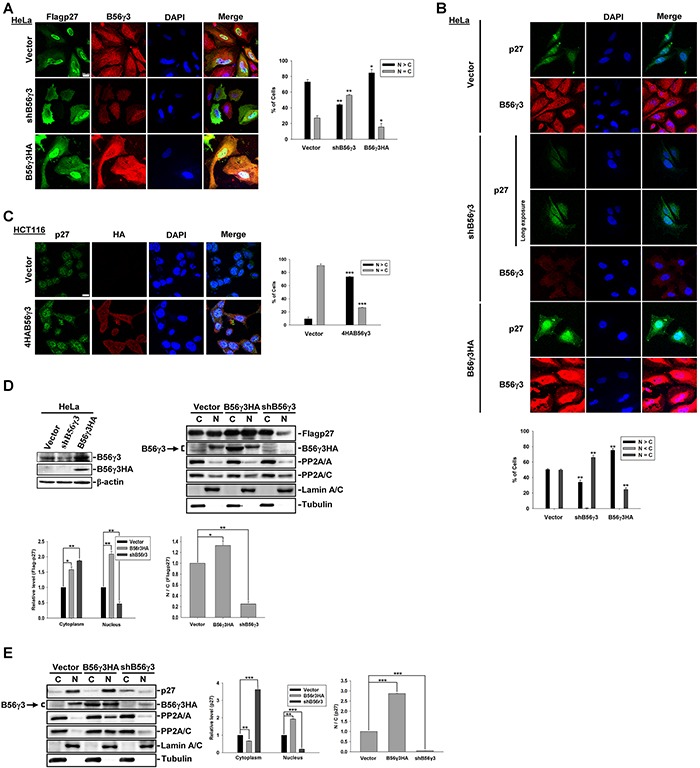
B56γ3 promotes nuclear localization of p27 **A.** The distribution of exogenous Flag-p27 was assessed in HeLa cells carrying vector only, B56γ3 overexpression, or B56γ3 knockdown. **B.** The distribution of endogenous p27 was assessed in HeLa cells carrying vector only, B56γ3 overexpression, or B56γ3 knockdown. One image acquired with long exposure setting was also shown for endogenous p27 in cells with B56γ3 knockdown. **C.** The distribution of endogenous p27 was assessed in HCT116 cells carrying vector only or B56γ3 overexpression. The indirect immunofluorescence was performed using mouse monoclonal anti-Flag or rabbit polyclonal anti-p27 antibodies in conjunction with Alexa488-conjugated anti-mouse or anti-rabbit secondary antibody, respectively. The endogenous B56γ3 were stained by rabbit anti-B56γ antibody in conjunction with Cy3-conjugated anti-rabbit secondary antibody. The nuclei were visualized by staining with DAPI. Scale bar, 20 μm. Cells with different staining patterns of p27 were scored as follows: predominantly nuclear (N>C), homogenously distributed in both nucleus and cytoplasm (N=C), and predominantly cytoplasmic (N<C). Graphs show quantitative analysis of Flag-p27 or endogenous p27 distribution in cells, and at least 100 cells were assessed from random fields. **D.** (Left) Lysates of HeLa cells with vector alone, B56γ3 overexpression, or B56γ3 knockdown were analyzed for B56γ3 expression using immunoblotting with antibodies specific for B56γ3 and β-actin. (Right) Lysates of HeLa cells with vector alone, B56γ3 overexpression, or B56γ3 knockdown were transfected with Flag-p27 were fractionated into cytoplasmic (C) and nuclear (N) fractions, and analyzed by SDS-PAGE and immunoblotting with antibodies specific for p27, FLAG, B56γ, PP2A/A, PP2A/C, lamin A/C and Tubulin. **E.** Lysates of HeLa cells with vector alone, B56γ3 overexpression, or B56γ3 knockdown were fractionated and analyzed as described above. The relative expression levels of Flag-p27 or endogenous p27 in the cytoplasmic and nuclear fractions were quantified by densitometry and normalized with tubulin or lamin A/C, respectively. The data shown are expressed as —fold expression level over that of vector-only cells, which was set as 1. Data shown are means ± S.D. of three experiments. The differences were assessed for statistical significance by Student's t test with p valu < 0.05(*), < 0.01 (**), or <0.001 (***).

To investigate the role of B56γ3 in the subcellular localization of p27, we transfected Flag-tagged p27 (mammalian expression construct of Flag-p27) into HeLa cells with vector only, stable HA-tagged B56γ3 (B56γ3HA) overexpression, or stably expressing B56γ3 shRNA (shB56γ3). We found that the distribution of Flag-p27 was predominantly nuclear in nearly 80 % of control cells carrying vector only, whereas p27 distribution became mainly ubiquitous throughout the entire cells when B56γ3 was knocked down (Figure 1A). As expected, B56γ3HA overexpression further enhanced the nuclear distribution of Flag-p27, and increased fluorescence intensity of Flag-p27 staining (Figure 1A). Similarly, the distribution of endogenous p27 showed predominantly nuclear localization in HeLa cells carrying vector only, whereas knockdown of B56γ3 resulted in ubiquitous distribution of p27 (Figure 1B). In addition, B56γ3 overexpression also significantly enhanced fluorescence intensity of the endogenous p27 staining (Figure 1B) in HeLa cells. B56γ3 overexpression also enhanced fluorescence intensity of the nuclear p27 staining in colon cancer cell line HCT116 (Figure 1C). To further confirm the results of fluorescence staining, we performed biochemical fractionation analysis of total cell lysates. Consistent with our previous finding that PP2A-B56γ3 increases p27 protein levels via downregulating phosphorylation of p27 at Thr187 [15], B56γ3 overexpression increased Flag-p27 protein levels (Figure 1D), whereas B56γ3 knockdown reduced p27 protein levels in both cytoplasmic and nuclear fractions (Figure 1D and 1E). In agreement with results of fluorescence staining (Figure 1A and 1B), results of biochemical fractionation analysis showed that B56γ3 overexpression increased ratio of nuclear relative to cytoplasmic fraction of p27, whereas in cells with B56γ3 knockdown, the ratio of nuclear relative to cytoplasmic fraction of both Flag-p27 and endogenous p27 were decreased compared to that of vector-only control cells (Figure 1D and 1E). Similar results were also found in esophageal cancer cell line TE1 (Supplementary Figure S1). Together, our data demonstrate that B56γ3 not only increases p27 levels but also promotes nuclear localization of p27.

### PP2A-B56γ3 selectively down-regulates phospho-p27 at Thr157, which is critical to subcellular localization of p27

**Figure 2:**
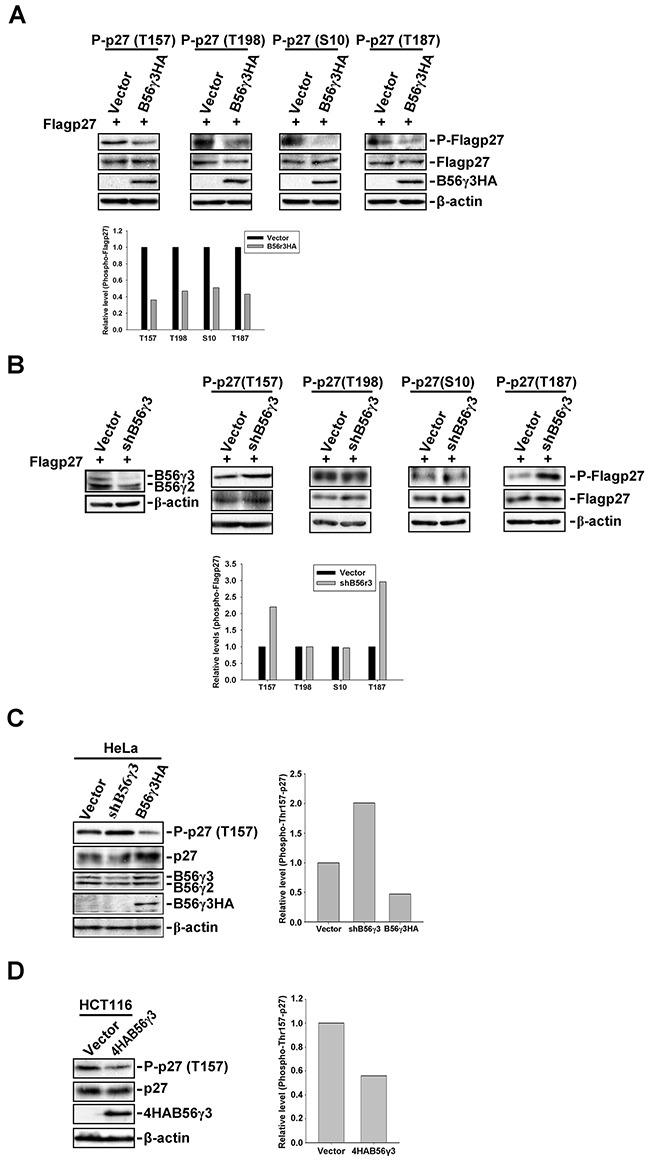
B56γ3 selectively regulates phosphorylation of p27 at Thr157 **A.** Lysates of HeLa cells stably overexpressing B56γ3HA or vector transfected with expression vector of Flag-p27 were analyzed by SDS-PAGE and immunoblotting with antibodies specific for phospho-p27 (Thr187), phospho-p27 (Thr157), phospho-p27 (Thr198), phospho-p27 (Ser10), total p27, HA, B56γ, and β-actin. **B.** Lysates of HeLa cells with vector only or B56γ3 knockdown transfected with expression vector of Flag-p27 were analyzed as described in (A). **C.** Lysates of HeLa with vector only, B56γ3 overexpression, or B56γ3 knockdown and **D.** lysates of HCT116 cells with vector only or B56γ3 overexpression were analyzed for levels of phospho-p27 (Thr157), p27, HA, B56γ3 and β-actin as described above. The amounts of protein applied to the gel were adjusted so that the lysates from different cells yielded similar total p27 levels detected on the blot. The relative expression level of each phospho-p27 were quantified by densitometry and normalized with total Flag-p27 or p27. The data shown are expressed as -fold expression level over that of vector control, which was set as 1. Data shown are from one representative experiment of at least two experiments with similar results.

Previous studies have shown that the subcellular localization of p27 was regulated by phosphorylation at several sites, including Ser10, Thr157 and Thr198 [21, 24]. We investigated whether B56γ3-containing PP2A can dephosphorylate p27 at these sites to regulate p27 nuclear localization. We transfected mammalian expression construct of Flag-p27 into HeLa cells with vector only or stable B56γ3HA overexpression and assessed the levels of phosphorylation at Ser10, Thr157, Thr198, and Thr187. In agreement with our previous finding, the level of phospho-Thr187 was reduced by B56γ3HA expression (Figure 2A) [15]. Surprisingly, levels of phospho-Ser10, -Thr157, and -Thr198 of p27 were also reduced when B56γ3HA was overexpressed (Figure 2A). Similar results were also found in NIH3T3 cells co-transfected with Flag-p27 and expression construct of 4HA-B56γ3 or vector (Supplementary Figure S2A). Nevertheless, only levels of phospho-Thr187 and Thr157, but not other sites, were increased as compared to that in control cells when we expressed Flag-p27 in HeLa cells with B56γ3 knockdown (Figure 2B). Similarly, we found that levels of phospho-Thr157 of endogenous p27 were decreased in HeLa, HCT116, HEK293T and TE1 cells overexpressing B56γ3 (Figure 2C, 2D, Supplementary Figure S1 and S2), but were increased in cells with B56γ3 knockdown (Figure 2C and Supplementary Figure S2). These results indicate that PP2A-B56γ3 holoenzymes may regulate the subcellular localization of p27 through dephosphorylating p27 at Thr157.

### B56γ3, but not B56α or B56γ1, selectively targets PP2A to regulate Thr157 dephosphorylation and nuclear localization of p27

**Figure 3:**
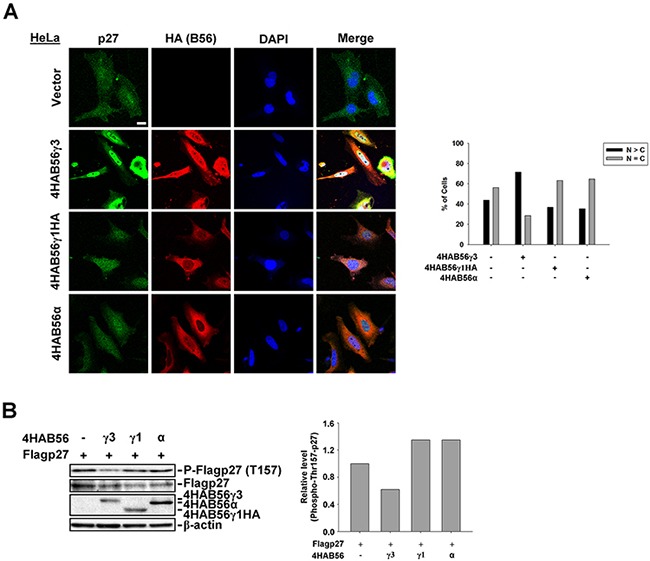
B56γ3, but not B56γ1 or B56α, promotes nuclear localization and decreases phospho-Thr157 of p27 **A.** HeLa cells were transfected with empty vector or expression vector of 4HA-B56α, 4HA-B56γ1HA, or 4HA-B56γ3. The distribution of p27 was assessed by indirect immunofluorescence using rabbit polyclonal anti-p27 antibody and Alexa488-conjugated anti-rabbit secondary antibody, and the exogenous HA-tagged B56 were stained by mouse anti-HA antibody in conjunction with Cy3-conjugated anti-mouse secondary antibody as described earlier. The nuclei were visualized by staining with DAPI. Scale bar, 20 μm. Cells with different staining patterns of p27 were scored as described earlier. Graphs show quantitative analysis of p27 distribution in cells, and at least 100 cells were assessed from random fields. **B.** Lysates of HeLa cells transfected with expression vector of Flag-p27 and empty vector or expression vector of 4HA-B56α, 4HA-B56γ1HA, or 4HA-B56γ3 were analyzed by SDS-PAGE and immunoblotting with antibodies as indicated. Data shown are from one representative experiment of at least two experiments with similar results.

To address whether other B56 regulatory subunits can also target PP2A to regulate phospho-Thr157 and subcellular localization of p27, we compared the effect on phospho-Thr157 and subcellular localization of p27 by co-expression of 4HA-B56γ3, 4HA-B56γ1HA, or 4HA-B56α with or without Flag-p27 into HeLa cells. As shown in Figure 3A, in contrast to results of co-expression of 4HA-B56γ3 and Flag-p27 that Flag-p27 is accumulated in the nucleus, the distribution of Flag-p27 was both in the nucleus and in the cytoplasm in cells co-transfected with Flag-p27 and 4HA-B56γ1HA or 4HA-B56α, which is similar to that of co-transfection with empty vector (Figure 3A). In addition, the level of phospho-Thr157 of Flag-p27 was decreased by 4HA-B56γ3 but not decreased by 4HA-B56γ1HA or 4HA-B56α (Figure 3B). Similarly, compared to that in cells stably expressing vector only, the level of phospho-Thr157 of Flag-p27 was decreased in NIH3T3 cells stably overexpressing B56γ3, but not by overexpressing B56γ1 (Supplementary Figure S3A). In addition, results of immunoprecipitation showed that levels of B56γ1 associated with p27 is less than that of B56γ3 (Supplementary Figure S3B), and B56γ1 overexpression only reduced the level of phospho-Thr187, but not phospho-Thr157, -Thr198 or -Ser10 (Supplementary Figure S4A). Together, these data demonstrated that B56γ3 selectively targets PP2A to regulate phospho-Thr157 and nuclear localization of p27.

### PP2A-B56γ3-regulated Thr157 dephosphorylation and nuclear localization of p27 does not result from down-regulating Akt activity

**Figure 4:**
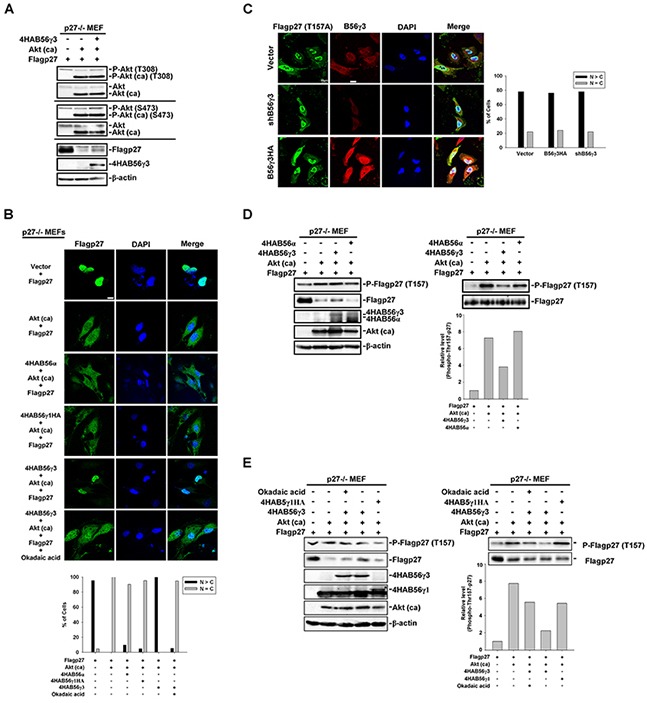
PP2A-B56γ3 counteracts Akt in regulating the subcellular localization of p27 independent of Akt down-regulation **A.** Lysates of p27−/− MEF cells transfected with expression vector of Flag-p27 and empty vector or expression vector of constitutive activated Akt (Akt (ca)) plus expression vector of 4HA-B56γ3 were analyzed by SDS-PAGE and immunoblotting with antibodies as indicated. **B.** p27−/− MEF cells were transfected with expression vector of Flag-p27 and empty vector or expression vector of constitutive activated Akt (Akt (ca)) plus expression vector of 4HA-B56α, 4HA-B56γ1HA, or 4HA-B56γ3 with or without treatment of okadaic acid. The distribution of Flag-p27 was assessed by indirect immunofluorescence using rabbit polyclonal anti-p27 antibody and Alexa488-conjugated anti-rabbit secondary antibody as described earlier. **C.** HeLa cells carrying vector only, B56γ3 overexpression or B56γ3 knockdown were transfected with expression vector of Flag-p27T157A and the distribution of Flag-p27T157A was assessed in by indirect immunofluorescence with mouse monoclonal anti-Flag antibody in conjunction with Alexa488-conjugated anti-mouse secondary antibody. The endogenous B56γ3 were stained by rabbit anti-B56γ3 antibody and Cy3-conjugated anti-rabbit secondary antibody. The nuclei were visualized by staining with DAPI. Scale bar, 20 μm. Cells with different staining patterns of Flag-p27T157A were scored as described earlier. Graphs show quantitative analysis of Flag-p27 or Flag-p27T157A distribution in cells, and at least 100 cells were assessed from random fields. Data shown are from one representative experiment of at least two experiments with similar results. **D–E**. Lysates of p27−/− MEFcells transfected with expression vector of Flag-p27 and empty vector or expression vector of constitutive activated Akt (Akt (ca)) plus expression vector of 4HA-B56α, 4HA-B56γ1HA, or 4HA-B56γ3 with or without treatment of okadaic acid were analyzed by SDS-PAGE and immunoblotting with antibodies as indicated. (D-E, right panel) The amounts of protein applied to the gel were adjusted so that the lysates from the different cells yielded similar total p27 levels detected on the blot. Data shown are from one representative experiment of at least two experiments with similar results.

It was shown that B56γ-containing PP2A negatively regulates Akt [29], which catalyzes phosphorylation of Thr157 of p27 [30]. To examine the possibility that B56γ3-mediated dephosphorylation of phospho-Thr157 of p27 was through down-regulating Akt activity, we co-transfected constitutively activated Akt (hereafter referred to as Akt (ca)), Flag-p27, and 4HA-B56γ3 or vector only into p27-null MEFs (p27−/− MEFs). We found that co-expression of 4HA-B56γ3 did not decrease but instead slightly increased phosphorylation of endogenous Akt at both Thr308 and Ser473 (Figure 4A). In addition, expression of Akt (ca) decreased p27 protein levels, but co-expression of Akt (ca) and 4HA-B56γ3 partially reversed the p27 protein levels (Figure 4A). In p27−/− MEFs transfected with Flag-p27 and empty vector, the subcellular localization of Flag-p27 was mainly nuclear (Figure 4B), but Flag-p27 became ubiquitously localized both in the nucleus and cytoplasm in cells co-transfected with Flag-p27 and Akt (ca) (Figure 4B). In contrast, when cells were co-transfected with Flag-p27, Akt (ca), and 4HA-B56γ3, Flag-p27 was accumulated in the nucleus, similar to that in cells transfected with Flag-p27 and empty vector (Figure 4B). The nuclear accumulation of Flag-p27 caused by co-expression of 4HA-B56γ3 was abolished by the PP2A-selective inhibitor okadaic acid (OA), indicating this regulation is mediated by PP2A catalytic activity. Consistent with the results of Figure 3, Akt (ca)-mediated ubiquitous distribution of Flag-p27 was not affected by co-transfection with 4HA-B56α or 4HA-B56γ1HA (Figure 4B). To further verify whether nuclear localization of p27 regulated by PP2A-B56γ3 holoenzymes is specifically through regulating phospho-Thr157 of p27, we transfected expression construct of Flag-tagged p27T157A (Flag-p27T157A), a phosphorylation defective mutant at Thr157, into HeLa cells with stably expressing vector only, B56γ3HA, or shB56γ3. The results showed that the distribution of Flag-p27T157A was predominantly nuclear in nearly 80 % of cells carrying vector only, and neither B56γ3HA overexpression nor B56γ3 knockdown affected the predominantly nuclear distribution of Flag-p27T157A (Figure 4C).

In agreement with the role of Akt in promoting p27 cytoplasmic localization by Thr157 phosphorylation of p27, results of immunoblottings showed that the phosphorylation of p27 at Thr157 was increased in cells co-transfected with Flag-p27 and Akt (ca) compared to that in cells transfected with Flag-p27 and vector only (Figure 4D). When cells were co-transfected with Flag-p27, Akt (ca), and 4HA-B56γ3, the level of phospho-Thr157 of p27 was decreased. In comparison with 4HA-B56γ3, the level of phospho-Thr157 of p27 was not decreased or moderately decreased when 4HA-B56α or 4HA-B56γ1HA was co-transfected, respectively, as compared to that in cells co-transfected with Flag-p27, Akt (ca), and vector (Figure 4D and 4E). In addition, decreased expression of phospho-Thr157-p27 mediated by B56γ3 was inhibited when cells were treated with OA (Figure 4E). Together, these data suggest that PP2A-B56γ3 counteracts Akt in regulating the subcellular localization of p27 via regulating p27 at Thr157, which is not attributed to down-regulating Akt activity.

### PP2A-B56γ3 directly dephosphorylates phospho-p27 at Thr157 *in vitro*

**Figure 5:**
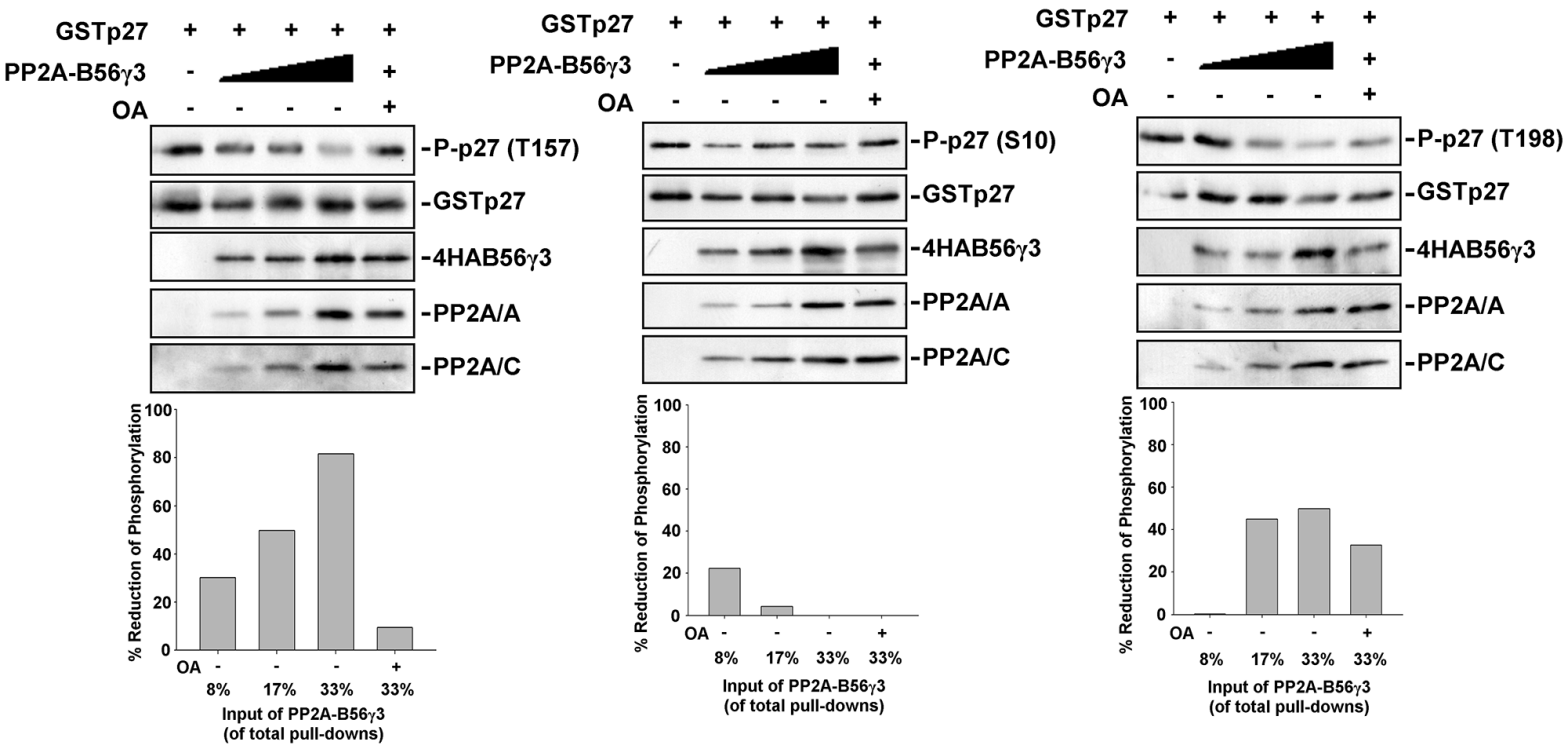
PP2A-B56γ3 selectively dephosphorylates p27 at Thr157 *in vitro* **A.** *In vitro* dephosphorylation reactions of phospho-p27 in the absence or presence of various amounts of B56γ3-containing PP2A complexes with or without 1 μM okadaic acid (OA) were carried out at 37°C for 30 min according to the procedure described under the “Materials and Methods”. Expression levels of phospho-p27 (Thr157), GST-p27, 4HA-B56γ3, and PP2A A and C subunits were detected by immunoblotting with antibodies specific for phospho-p27 (Thr157), phospho-p27 (Ser10), phospho-p27 (Thr198), GST, HA, PP2A/A and PP2A/C. The levels of p27 phosphorylation were quantified by densitometry and normalized with total p27. Levels of control reactions with no addition of PP2A-B56γ3 complexes were set as 100 %. Data expressed as percentages of reduction of phospho-p27 in individual reactions in the presence of PP2A-B56γ3 complexes with or without OA. Data shown are from one representative experiment of at least two experiments with similar results.

We found that PP2A-B56γ3 can counterbalance Akt phosphorylation of p27 at Thr157 (Figure 4), and we previously showed B56γ3-containing PP2A directly interacts with p27 and dephosphorylates p27 at Thr187 [15]. Next, we examined whether PP2A-B56γ3 holoenzymes can catalyze dephosphorylation of phospho-p27 at Thr157 *in vitro*. We performed *in vitro* dephosphorylation assay for bacterially expressed recombinant GST-p27 which was phosphorylated by Akt *in vitro* in the presence of various amounts of purified PP2A-B56γ3 holoenzymes with or without OA. Results of *in vitro* dephosphorylation showed that PP2A-B56γ3 holoenzymes catalyzed dephosphorylation of phospho-Thr157 in a dose-dependent manner (Figure 5), and the phosphorylation of Thr157 was restored in the presence of the PP2A-selective inhibitor OA (Figure 5), indicating that dephosphorylation of Thr157 by PP2A-B56γ3 holoenzymes *in vitro* was specifically carried out by PP2A catalytic activity. In contrast, dephosphorylation of phospho-Ser10 and of phospho-Thr198 by PP2A-B56γ3 holoenzymes was not dose-dependent and inefficient, resulting in maximal reduction of phosphorylation of Ser10 and Thr198 at approximately 20% and 40 %, respectively, as compared to 80% at phospho-Thr157 (Figure 5). Consistently, *in vitro* dephosphorylation assay also showed that PP2A-B56γ1 cannot efficiently catalyze dephosphorylation of phospho-Thr157, phospho-Ser10 and phospho-Thr198 (Supplementary Figure S4B).

### Mapping of the interacting domains between p27 and B56γ3 reveals both the N-terminal and C-terminal domains of p27 and a domain in the C-terminus of B56γ3 are required for interaction between p27 and B56γ3

**Figure 6:**
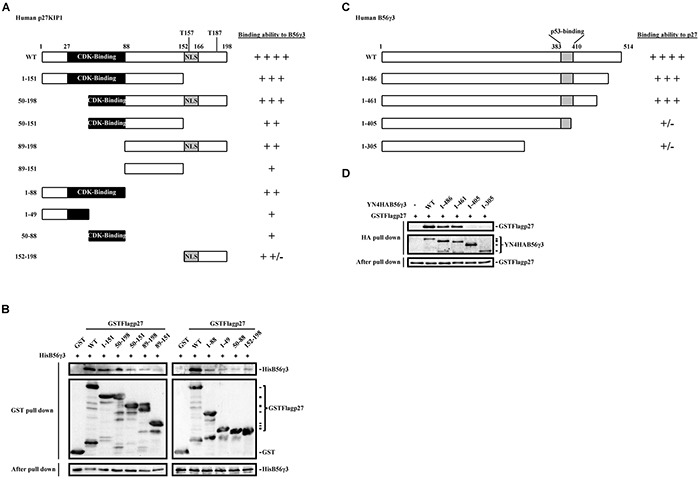
Interaction domain mapping of p27 and B56γ3 reveals two domains of p27, residues 1-88 and residues 152-198, responsible for B56γ3 interaction, and a C-terminal domain of B56γ3, residues 406-461, involved in binding to p27 **A.** Schematic diagrams show serial deletion p27 proteins with different binding ability with B56γ3 as indicated. **B.** *In vitro* pulldown analysis was carried out following incubating 2 μg of recombinant GST, GST-p27 WT or GST-p27 serial deletion proteins with 3 μg of recombinant His-B56γ3 at 4°C for 4 h. GST-pulldowns were then analyzed by SDS-PAGE and immunoblotting with antibodies for GST and B56γ3. **C.** Schematic diagrams show serial deletion B56γ3 proteins with different binding ability with p27 as indicated. **D.** *In vitro* pulldown analysis was carried out following incubating 300 μg of lysates of NIH3T3 cells transfected with empty vector or expression vector of YN-4HA-B56γ3 WT or YN-4HA-B56γ3 (serial deletion mutants) with 0.5 μg recombinant GST-FLAG-p27 WT at 4°C for 4 h. HA-pulldowns were then analyzed by SDS-PAGE and immunoblotting with antibodies for GST and HA. Ten percent of mixed recombinant proteins after pulldown analysis were analyzed in parallel, serving as a loading control.

We have previously used co-immunoprecipitation and *in vitro* pulldown assay to demonstrate the direct interaction of B56γ3 and p27 [15]. Here, we further investigated the interacting domains between B56γ3 and p27. We created a series of GST-fused deletion mutants of p27 encompassing residues 1-151, 50-198, 50-151, 89-198 or 89-151 (Figure 6A). By *in vitro* pulldown assay, we found that the association of GST-p27 with His-B56γ3 proteins was significantly reduced when the N-terminal domain encompassing residues 1-88 or the C-terminal domain encompassing residues 152-198 of p27 was deleted (Figure 6B, left). Reciprocally, we mapped the p27-interacting domain of B56γ3 by employing a series of B56γ3 deletion mutants, encompassing residues 1-486, 1-461, 1-405 or 1-305 fused with a YFP N-terminal fragment and 4xHA tag (YN-4HA-B56γ3) (Figure 6C). By *in vitro* pulldown assay, we found that the association of YN-4HA-B56γ3 with GST-p27 proteins was abolished when the domain encompassing residues 406-461 of B56γ3 was deleted (Figure 6D). These data indicate that an N-terminal and a C-terminal domains of p27, residues 1-88 and residues 152-198, cooperate to accomplish the association of p27 with B56γ3, and at least a C-terminal domain of B56γ3, residues 406-461, mediates the binding of B56γ3 with p27.

### PP2A-B56γ3 inhibits CDK2 activity

**Figure 7:**
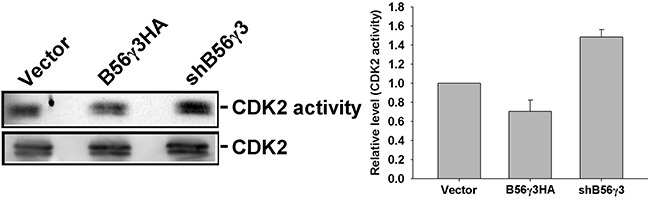
PP2A-B56γ3 inhibits CDK2 activity CDK2 was immunoprecipitated from HeLa cells carrying vector only, B56γ3 overexpression or shB56γ3 by anti-CDK2 antibody. CDK2 kinase activities were assessed by using Histone H1 as substrate in the presence of [γ-^32^P]-ATP and kinase reactions were performed according to the procedure described under the “Materials and Methods”. Subsequently, reaction products were resolved by SDS-PAGE and analyzed by autoradiography. Relative CDK activities were quantified by densitometry and normalized with total CDK2 protein levels which were determined by immunoblotting. Representative immunoblotting data shown are from one representative experiment and quantified data of relative CDK activities shown are means ± S.D. of at least three experiments with similar results.

Since p27 is a CDK2 inhibitor and PP2A-B56γ3 increases both levels and nuclear localization of p27, we further investigated whether PP2A-B56γ3 inhibits CDK2 activity. We measured the CDK2 activity in HeLa cells with vector only, stable B56γ3HA overexpression, or stably expressing shB56γ3. Compared to that in cells expressing vector only, CDK2 activity was decreased in cells with stable B56γ3HA overexpression, but increased in cells with B56γ3 knockdown (Figure 7). Taken together, our data indicate that the PP2A-B56γ3 holoenzyme catalyzes dephosphorylation of both phospho-Thr157 and Thr187 of p27 to up-regulate levels and nuclear localization of p27, resulting in down-regulating CDK2 activity.

### Levels of B56γ are positively correlated with levels of p27, but are only positively correlated with nuclear levels of p27 in non-tumor parts of human colon cancer tissue specimens

**Figure 8:**
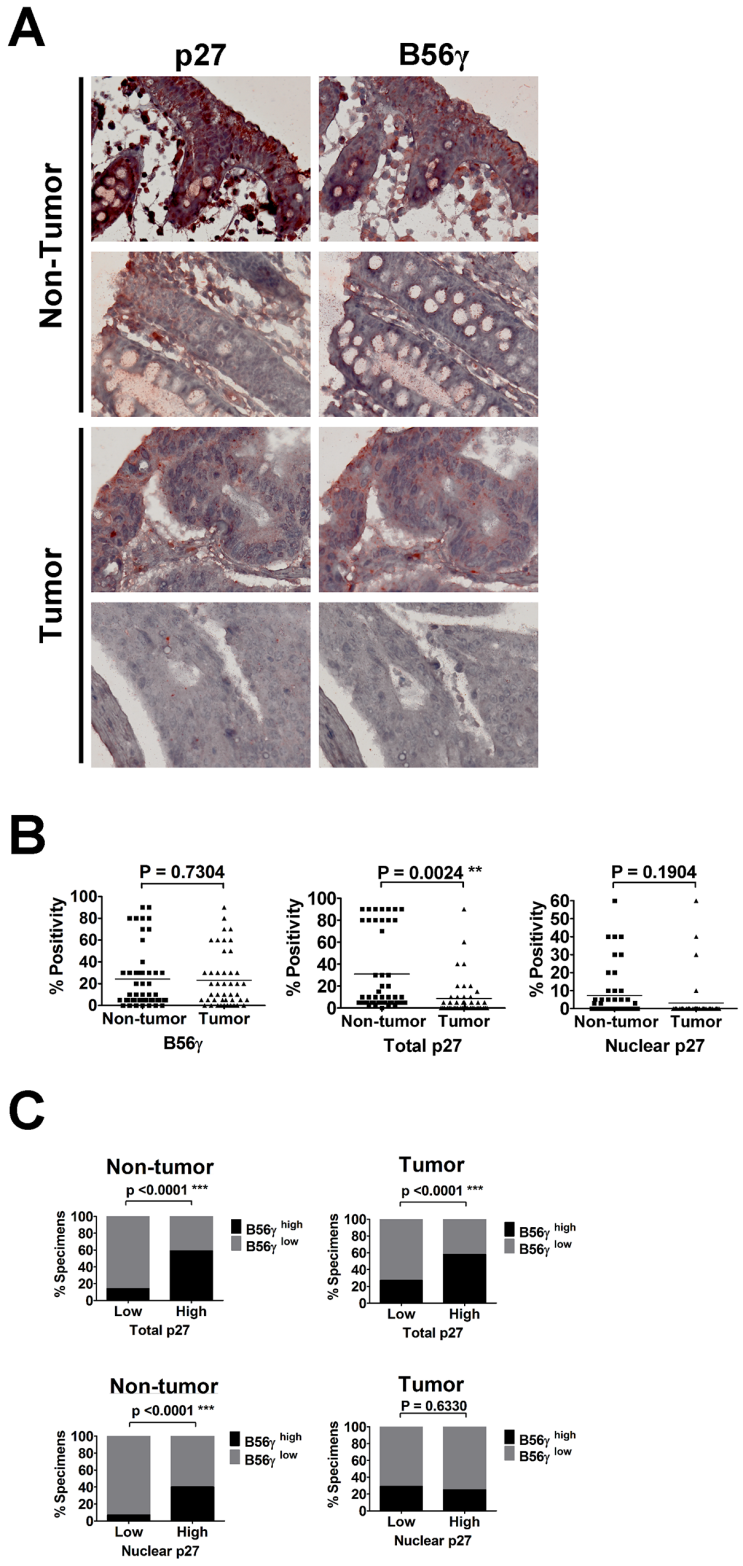
IHC analysis of p27 and B56γ proteins in human colon cancer specimens reveals positive correlation between levels of p27 and B56γ **A.** Representative results of IHC analysis of p27 and B56γ protein levels in tumor/non-tumor tissues of 50 human colon cancer specimens are shown. **B.** Quantitative analysis of positive staining of nuclear p27, total p27, and B56γ in both tumor and non-tumor parts of 50 paired human colon cancer specimens is shown. **C.** Upper graphs show percentages of specimens with low (or not observed) or high total p27 expression in which B56γ expression was high or low (or not observed) in non-tumor or tumor parts of the paired colon cancer specimens. Lower graphs show percentages of specimens with low (or not observed) or high nuclear p27 expression in which B56γ expression was high or low (or not observed) in non-tumor or tumor parts of the paired colon cancer specimens. Positive correlations were noted between total p27 and B56γ in both non-tumor and tumor parts (P < 0.0001***), and between nuclear p27 and B56γ in non-tumor parts (P < 0.0001***).

Since both B56γ and p27 mainly function as a tumor suppressor, to further investigate the association between B56γ and p27 in tumorigenesis, we studied the correlation between p27 nuclear localization and expression of B56γ in human cancer specimens. Given that p27 mislocalization was found to associate with colon cancer [22, 31, 32], we evaluated the expression of p27 and B56γ in a tissue microarray of 50 human colon tissue specimens by immunohistochemistry (IHC) staining (Figure 8A). Although results of IHC on these colon tissue specimens showed no association of expression of B56γ or nuclear p27 levels with non-tumor versus tumor parts of colon cancer tissue specimens, reduced total p27 levels were found in tumor parts as compared with non-tumor parts of the colon cancer tissue specimens (Figure 8B). In addition, we found that high levels of B56γ was detected in 16 (59%) of 27 and 7 (58%) of 12 specimens with high total p27 expression in non-tumor and tumor parts, respectively, but high levels of B56γ was only detected in 3 (14%) of the 21 and 9 (27%) of 33 specimens with low total p27 expression in non-tumor and tumor parts, respectively (Figure 8A and C upper panel), consistent with our previous finding that B56γ3 positively regulates p27 levels [15]. In addition, IHC staining revealed that high B56γ was detected in 8 (40%) of 20 specimens with high nuclear p27 expression, but it was only detected in 2 (7%) of the 27 specimens with low nuclear p27 expression in non-tumor parts of colon cancer specimens (Figure 8A and 8C, lower panel). However, the positive correlation between levels of B56γ and nuclear p27 was abolished in tumor parts of colon cancer tissue specimens, which showed that high levels of B56γ was detected in 1 (25%) of 4 specimens with high nuclear p27 expression and 5 (29%) of 17 specimens with low nuclear p27 expression (Figure 8A and 8C, lower panel). Together, these results indicate that B56γ3 expression is positively associated with expression of total p27 in both non-tumor and tumor parts of colon cancer tissue specimens and is only correlated with nuclear p27 levels in non-tumor parts of colon cancer tissue specimens.

## DISCUSSION

We have identified B56γ3 as a regulatory subunit targeting the PP2A holoenzyme to regulate the subcellular localization of p27 as evidenced by down-regulation of cytoplasmic localization and phosphorylation of p27 at Thr157 by expression of exogenous B56γ3 gene, and increased cytoplasmic localization and phosphorylation of p27 by silencing endogenous B56γ3 gene expression. In addition, B56γ3 targeting the PP2A holoenzyme to p27 is more selective toward phosphorylated Thr157 than the other two localization-regulatory sites, Ser10 and Thr198. Further, our data demonstrate that PP2A-B56γ3 regulating the distribution and phosphorylation of p27 is not through down-regulating Akt, an upstream kinase of p27 regulating Thr157 phosphorylation of p27 and outcompeted a myristoylated constitutively active Akt (Aktca). Furthermore, results of IHC staining of the colon tissue specimens showed that the expression levels of B56γ are positively correlated with the levels of nuclear p27 only found in normal parts of the specimens, but not tumor parts. Moreover, we identified two B56γ3-binding domains in p27, residues 1-88 and residues 152-198. On the other hand, we identified a domain encompassing residues 406-461 within B56γ3 mediating B56γ3 binding with p27. Our findings demonstrate that PP2A-B56γ3 selectively dephosphorylates p27 at Thr157 to regulate subcellular localization of p27, which is dysregulated in tumorigenesis.

The cytoplasmic mislocalization of p27 has been found in some types of cancer, such as breast, colon, ovarian, thyroid and esophageal cancers [22], which is associated with poor prognosis, high tumor grade or metastasis [22]. Activation of K-RAS, the hyperactive PI3K/Akt signaling pathway, SGK1 overexpression, or highly activated RSK, sequestrates p27 to the cytoplasm through regulating the phosphorylation of p27 at Ser10, Thr157, or Thr198 [20, 22, 33–36]. Furthermore, it was postulated that both phosphorylation of p27 at Thr157 and Thr198, but not Ser10, are required to binding to 14-3-3, which sequestrates p27 in the cytoplasm [23, 37]. On the other hand, the role of protein phosphatase in regulating subcellular localization of p27 was largely unknown until now. We showed that depletion of PP2A-B56γ3 facilitates p27 cytoplasmic localization (Figure 1). Moreover, our data of IHC suggest that dysregulation of PP2A-B56γ3 in control p27 localization may occur in tumorigenesis of colon. Why PP2A-B56γ3 becomes dysregulated in regulating p27 localization in tumorigenesis? Reports of somatic mutations of the PPP2R1A and PPP2R1B genes, which encode the α and β isoforms of the structural PP2A/A subunit, were found to be associated with various human cancers, including lung cancer, colon cancer, breast cancer, skin cancer and ovarian cancer [38–41]. Importantly, several mutations of the PP2A/A subunit associated with human cancers resulted in loss of ability to form complex with the regulatory B subunit and, especially, a mutation G90D alters Aβ ability to interact with B56γ subunits [40]. In addition, a recent report demonstrates that several mutations of B56γ found in human cancer samples abrogate B56γ interaction with the AC core enzyme of PP2A [42]. Therefore, it is possible that mutations of A or/and B56γ3 subunits causing failure in formation of a functional PP2A-B56γ3 holoenzyme complex may contribute to the dysregulation of p27 subcellular localization. Additionally, it is possible that mutations of B56γ may impair its interaction with substrates, such as p27, which were found in the case of interacting with p53 [14]. Nevertheless, the role of mutations of A or B56γ3 subunits in dysregulating p27 subcellular localization needs further investigation.

Several lines of evidence support that PP2A-B56γ3 can facilitate p27 nuclear localization through direct dephosphorylation of p27 at phospho-Thr157. Firstly, overexpression of B56γ3 decreased phosphorylation at Thr157, whereas knockdown of B56γ3 increased phosphorylation at Thr157 (Figure 2). Secondly, overexpression of B56γ3 did not down-regulate, but instead slightly up-regulate the phosphorylation at both Ser473 and Thr308 of Akt, the major upstream kinase for Thr157 (Figure 4). Thirdly, PP2A-B56γ3 can still dephosphorylate phospho-Thr157 and promote nuclear localization of p27 in the presence of constitutively activated Akt (Figure 4). Fourthly, B56γ3 overexpression did not impact phosphorylation (phospho-S255/T256) of SGK, which can also phosphorylate Thr157 of p27 (Supplementary Figure S5). Lastly, PP2A-B56γ3 can catalyze dephosphorylation of phospho-Thr157 at a dose-dependent manner *in vitro* (Figure 5).

It is known that the B regulatory subunits regulate the substrate specificity of the PP2A holoenzyme. We found that B56γ3, but not B56γ1 or B56α, significantly promotes dephosphorylation at Thr157 and nuclear localization of p27 (Figure 3). Sequence analysis indicates that the three isoforms show great similarity (86%) within most of the amino acid sequence, but they differ markedly at the C-terminus. Although B56γ1 and B56γ3 are splice isoforms of the B56γ family, only B56γ3 can promote dephosphorylation at Thr157 and nuclear localization of p27, demonstrating a highly isoform-specific regulation on substrate dephosphorylation. B56γ1 and B56γ3 share the first 441 amino acids, but have a distinct c-terminal domain. Results of mapping of p27-interacting domain of B56γ3 revealed that the domain encompassing residues 406 to 461 mediates B56γ3 binding to p27 (Figure 6). Among 56 amino acid residues (406 to 461) of the p27-binding domain of B56γ3, B56γ1 shares 26 amino acids and B56α shares total 11 amino acids of the first 26 amino acids with B56γ3, but varies greatly in the rest of the sequence within the 56 residues. The sequence similarity in the p27-interacting domain between B56γ1 and B56γ3 explains why both B56γ3 and B56γ1 can associate with p27, but B56γ1 binds much less p27 as compared to B56γ3 (Supplementary Figure S3). The results indicate that the distinct C-terminal domain of B56γ3 determines the specificity of catalyzing dephosphorylation at Thr157 of p27. In addition, we identified two domains of p27, residues 1-88 and residues 152-198, crucial for its association with B56γ3 (Figure 6). The domain, residues 152-198, is near the C-terminus and includes Thr157 and Thr187, which are two target sites of PP2A-B56γ3. Intriguingly, the N-terminal domain, residues 1-88, was shown to include a cyclin-CDK-binding region, implicating that B56γ3 and CDK may compete for binding to the same or close domain in p27 to regulate the activity of p27. It will be interesting to investigate whether Akt also interacts with the overlapping region as CDK and PP2A-B56γ3 do. Furthermore, B56γ3 overexpression can reduce phosphorylation levels of Ser10, Thr157, Thr187, and Thr198, whereas results of B56γ3 knockdown and *in vitro* dephosphorylation assays demonstrated that PP2A-B56γ3 preferentially dephosphorylates p27 at Thr157 and Thr187, but not Ser10 or Thr198. It is possible that dephosphorylation at Thr157 may disrupt or weaken 14-3-3 binding, which subsequently facilitates dephosphorylation of p27 at phospho-Thr198 and phospho-Ser10. The competition between 14-3-3 and PP2A in associating phospho-p27 is very similar to that found in dephosphorylation of the pro-apoptotic BAD, in which PP2A competes with 14-3-3 to dephosphorylate phospho-Ser112, subsequently facilitating dephosphorylation of phospho-Ser136 [43].

Decreases in p27 levels have been found in early stage of colon cancer [44], and were correlated with increased risk of disease recurrence and death [44–46]. Some studies showed that high nuclear p27 scores are associated with improved outcome [44, 47]. Moreover, some studies showed that reduced nuclear p27 levels are correlated with the advanced, metastatic stage, but not the earlier stage of colon cancers [31, 32, 44, 48]. Our data also demonstrated that the levels of total p27 in tumor parts are decreased compared to that in non-tumor parts of colon cancer specimens (Figure 8B). In agreement with our previous finding [15], we found that total p27 levels are correlated with the levels of B56γ3 in these colon cancer specimens (Figure 8C). In addition, we found correlation between levels of nuclear p27 and B56γ only in the normal parts, but not in the tumor parts of these specimens (Figure 8C). Based on our and others' findings, we propose that dysregulation of PP2A-B56γ3 in regulating levels and subcellular localization of p27 may contribute to colon cancer progression.

p27 induction was recently demonstrated to associate with mTOR inactivation in gerosuppression, suppression of conversion from cell cycle arrest to senescence stimulated by growth signaling in contact-inhibited cells [49, 50]. Furthermore, p27 was found to up-regulate expression of microRNA (miR) 223 to regulate cell cycle exit following contact inhibition [51]. On the other hand, mTOR activation was required for conversion from cell cycle arrest to senescence [52] and was shown to inhibit PP2A activity [53, 54]. We previously showed that PP2A-B56γ3 regulates the G1/S transition by dephosphorylating phospho-Thr187 and stabilizing p27 levels [15]. In addition, we found that higher levels of contact inhibition-induced p27 proteins were maintained in cells overexpressing B56γ3 after replating cells in lower density compared to that of control cells [15]. Together, in addition to its tumor suppressive activity, PP2A-B56γ3 may also participate in regulating gerosuppression by regulating levels and nuclear localization of p27 and mTOR inactivation.

## MATERIALS AND METHODS

### Antibodies, reagents, and DNA constructs

Antibodies employed include mouse monoclonal anti-HA tag (6E2), rabbit monoclonal anti-HA tag (C2F9), anti-phospho-Akt (Thr308) (C31E5), anti-phospho-Akt (Thr473) (D9E) and anti-lamin A/C from Cell Signaling; anti-PP2A/A (C-20), anti-CDK2 (M2) and anti-p27 (C19) from Santa Cruz; anti-Akt monoclonal antibody, anti-PP2A/C and anti-p27 from BD Transduction Laboratories; anti-tubulin, clone DM1A, and anti-phospho-Kip1 (Thr187) from Upstate; anti-β-actin and anti-FLAG (M2) from Sigma; anti-HA tag monoclonal antibody (HA.11) from Covance; anti-GST antibody and Glutathione-Sepharose from GE Healthcare; anti-phospho-p27/Kip1 (Thr157) and anti-phospho-p27/Kip1 (Thr198) from R&D Systems; anti-phospho-p27 (Ser10) from Invitrogen. Alexa 488-conjugated secondary antibodies were from Invitrogen. Secondary antibodies, HRP-conjugated anti-mouse IgG and HRP-conjugated anti-rabbit IgG were from Jackson ImmunoResearch. HRP-conjugated anti-goat IgG was from Santa Cruz. Serine/threonine phosphatase inhibitor okadaic acid was from Alexis Biochemicals. Protease inhibitors, phenylmethylsulfonyl fluoride, aprotinin, and leupeptin, were from Sigma. 4′,6-Diamidino-2-phenylindole dihydrochloride (DAPI) was from Molecular Probes. Lipofectamine 2000 was from Invitrogen. Digitonin and Histone H1 were from Calbiochem. The polyclonal antibody against B56γ (γ2 and γ3) was generated as described previously [55]. The constitutively active Akt with an N-terminal Src myristoylation signal sequence was prepared by PCR using a template pECE-myr-Akt and subsequently cloned into pcDNA3.1 vector [56]. The pBMNIRES-EGFP-FLAG-p27 was a gift from Dr. Carlos Arteaga [28]. The pEF-BOS-Flagp27 and pcDNA3.1-Flagp27T157A were gifts from Dr. Yoshihiro Yoneda [23]. The deletion mutants of N-terminal FLAG-tagged human p27 were prepared by PCR using the pGEX4T-1-Flagp27 WT as a template and were subsequently cloned into the bacterial expression vector pGEX4T-1 (GE Healthcare). The full-length human B56γ1 and B56α cDNA with HA-tag were prepared by PCR using the pCEP4-B56γ1 and pCEP4-B56α, respectively, as a template and were subsequently cloned into the pcDNA3.1 and the retroviral pMSCVpuro vector. The expression construct of YN-4HA-B56γ3 WT was established by cloning cDNA of 4HA-B56γ3 WT into pcDNAI-YFPN vector (kind gift of Dr. Catherine Berlot). The expression constructs of YN-4HA-B56γ3 serial deletion mutants were established by cloning cDNA of HA-tagged deletion mutant of B56γ3 (residues 1-486, residues 1-461, residues 1-405, or residues 1-305) which was obtained by PCR-based method into pcDNAI-YFPN vector.

### Cell culture, cell lines, and transfection

NIH3T3 and HeLa were cultured as previously described [15]. P27−/− MEFs were kindly provided by Dr. Elizabeth Yang [57]. MEFs and HEK293T were cultured in DMEM supplemented with 10% fetal bovine serum (FBS). HCT116 (ATCC) was cultured in Dulbecco's Modified Eagle Medium: Nutrient Mixture F-12 (DMEM/F12) supplemented with 10% FBS. A polyclonal pool of cells stably expressing HA-tagged B56γ3, B56γ1, B56α, vector only, B56γ3-specific shRNA, or shLuc was created by retroviral or lentiviral infection and subsequent drug selection (usually more than 80% survival) as previously described [58]. Transient transfection experiments were carried out by Lipofectamine 2000 according to standard procedures.

### Subcellular fractionation and western blotting

The subcellular fractionation was performed according to the procedures described previously [59] with a few modifications. The cells were washed in PBS and incubated in transport buffer as described previously [59], containing 40 μg/ml digitonin and protease and phosphatase inhibitor (1 mM PMSF, 10 μg/ml aprotinin, 10 μg/ml leupeptin, 5 mM EGTA, 5 mM EDTA, 10 mM NaF, 500 nM okadaic acid, and 1 mM Na_3_VO_4_) at 4°C for 5 min. The supernatant was collected as cytoplasmic fraction. The nuclear fraction was obtained by sonication (15 % amplitude, six times, 10 sec each at 10 sec intervals) of the pellet in transport buffer. Whole cell lysates for Western blotting were prepared in radio immunoprecipitation assay buffer as described previously [60] with freshly added protease and phosphatase inhibitors as described above. Whole cell lysates and the cytoplasmic and nuclear extracts were then mixed with 4X SDS sample buffer and subjected to SDS-PAGE and Western blotting. Immunoblots were developed using enhanced chemiluminescence. Results were quantified by densitometry using Alpha Innotech AlphaImager.

### Immunofluorescence and microscopy

The immunofluorescence was performed according to the procedures described previously [15]. Following immunostaining with the antibodies as indicated, DAPI was applied to stain the nuclei. Results were visualized by fluorescence microcopy (ZEISS, Axio Observer Z1) at 400X magnification or confocal microcopy (Olympus, FV1000) at 600X magnification. Quantification of fluorescence images for subcellular distribution of immunostained proteins was performed by scoring at least 100 cells on slides by dividing staining patterns as predominantly nuclear (N>C), homogenously distributed in an entire cell (N≈C), and predominantly cytoplasmic (N<C).

### *In Vitro* p27 dephosphorylation analysis

The *in vitro* p27 dephosphorylation analysis was performed according to the procedures described before [15].

### *In vitro* CDK2 activity assay

The *in vitro* CDK2 activity assay was performed according to the procedures described previously [61].

### *In vitro* pull down assay

The recombinant His-B56γ3, GST, GST-Flag-p27 WT, or serial deletion mutants GST-Flag-p27 proteins were prepared by following the protocols described before [58]. For mapping of the p27-interacting domain within B56γ3, 300 μg of lysates of NIH3T3 cells transfected with empty vector or expression vector of YN-4HA-B56γ3 WT or YN-4HA-B56γ3 (serial deletion mutants) were incubated with 0.5 μg recombinant GST-FLAG-p27 WT. Analysis of direct interactions of B56γ3 and p27 *in vitro* was performed according to the procedures described previously [15].

### Clinical specimens

The tissue arrays of human colon tissue specimens (50 cases/100 cores) were purchased from US Biomax (catalog number BC05118b).

### Immunohistochemistry (IHC)

Paraffin sections of tumor blocks were stained using anti-p27 antibody (C-19, Santa Cruz) and anti-B56γ antibody. Sections were counterstained with hematoxylin. The degree and localization of p27 and B56γ staining were scored independently by two pathologists (H.W.Tsai and C.J. Yen).

### Statistical analysis

Data of levels of B56γ and p27 in IHC were analyzed by unpaired, two-sided student's t test using Prism4 (Graphpad software).

## SUPPLEMENTARY FIGURES


