# Supplementary material for: The B56γ3 regulatory subunit-containing protein phosphatase 2A outcompetes Akt to regulate p27KIP1 subcellular localization by selectively dephosphorylating phospho-Thr157 of p27KIP1
Source: Oncotarget. 2015 Dec 14;7(4):4542–58. doi: 10.18632/oncotarget.6609 (PMC4826225; doi:10.18632/oncotarget.6609)
Supplement: Supplementary file 1 [file oncotarget-07-4542-s001.pdf]

SUPPLEMENTARY FIGURES

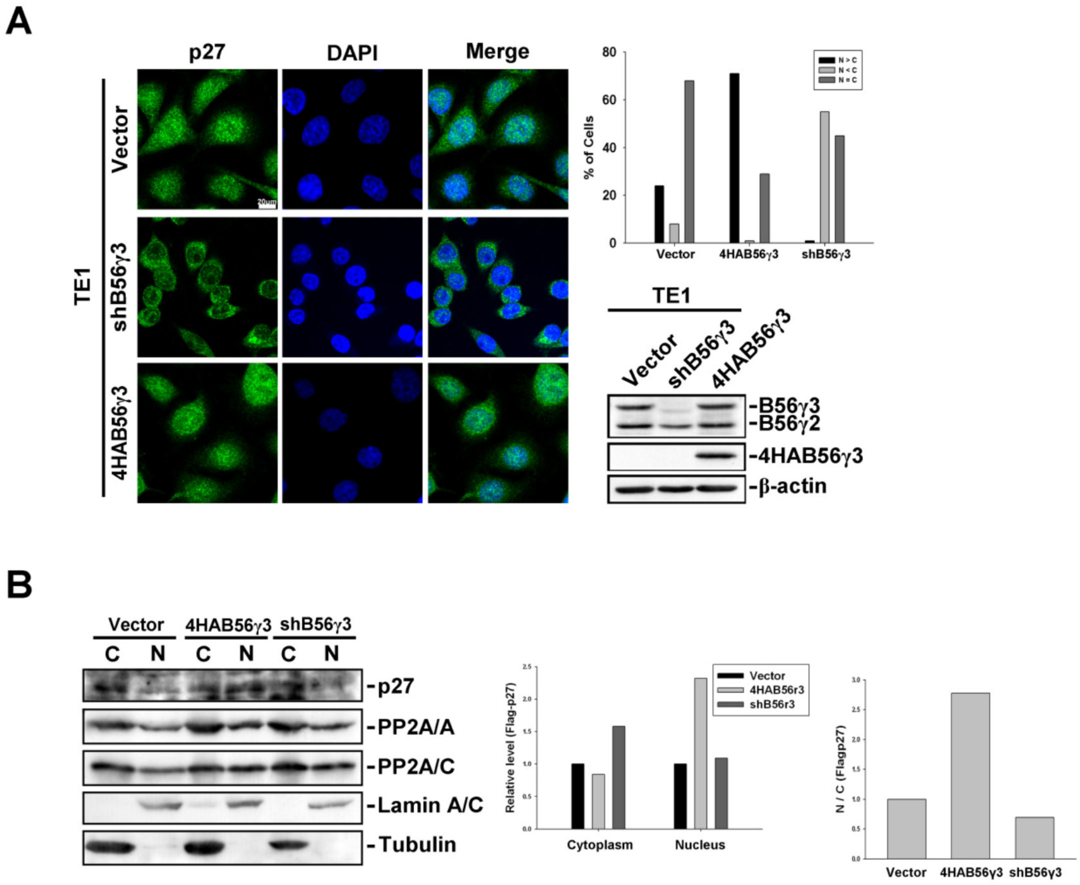

Supplementary Figure S1: B56γ3 promotes nuclear localization and decreases phospho-Thr157 of p27 in TE1 and HCT116 cells. The distribution of endogenous p27 was assessed in TE1 **A.** carrying vector only, B56γ3 overexpression or B56γ3 knockdown by indirect immunofluorescence with rabbit polyclonal anti-p27 antibodies in conjunction with Alexa488-conjugated anti-rabbit secondary antibody. The expression of exogenous B56γ3 in HCT116 cells was stained by mouse anti-HA antibody and Cy3-conjugated anti-mouse secondary antibody. The nuclei were visualized by staining with DAPI. Scale bar, 20 μm. Cells with different staining patterns of p27 were scored as follows: predominantly nuclear (N > C), homogenously distributed in both the nucleus and cytoplasm (N=C), and predominantly cytoplasmic (N < C). Graphs show quantitative analysis of p27 distribution in cells, and at least 100 cells were assessed from random fields. The expression level of B56γ3 in TE1 cells with vector alone, B56γ3 overexpression, or B56γ3 knockdown were analyzed by immunoblotting with antibodies for B56γ, HA and β-actin. **B.** Lysates of TE1 cells with vector alone, B56γ3 overexpression, or B56γ3 knockdown were fractionated into the cytoplasmic **C.** and nuclear fractions (N), and analyzed by immunoblotting with antibodies specific for p27, PP2A/A, PP2A/C, lamin A/C and Tubulin. The relative expression levels of endogenous p27 in the cytoplasmic and nuclear fraction were quantified by densitometry and normalized with tubulin or lamin A/C, respectively. The data shown are expressed as –fold expression level over that of vector cells, which was set as 1. Data shown are from one representative experiment of at least two experiments with similar results.

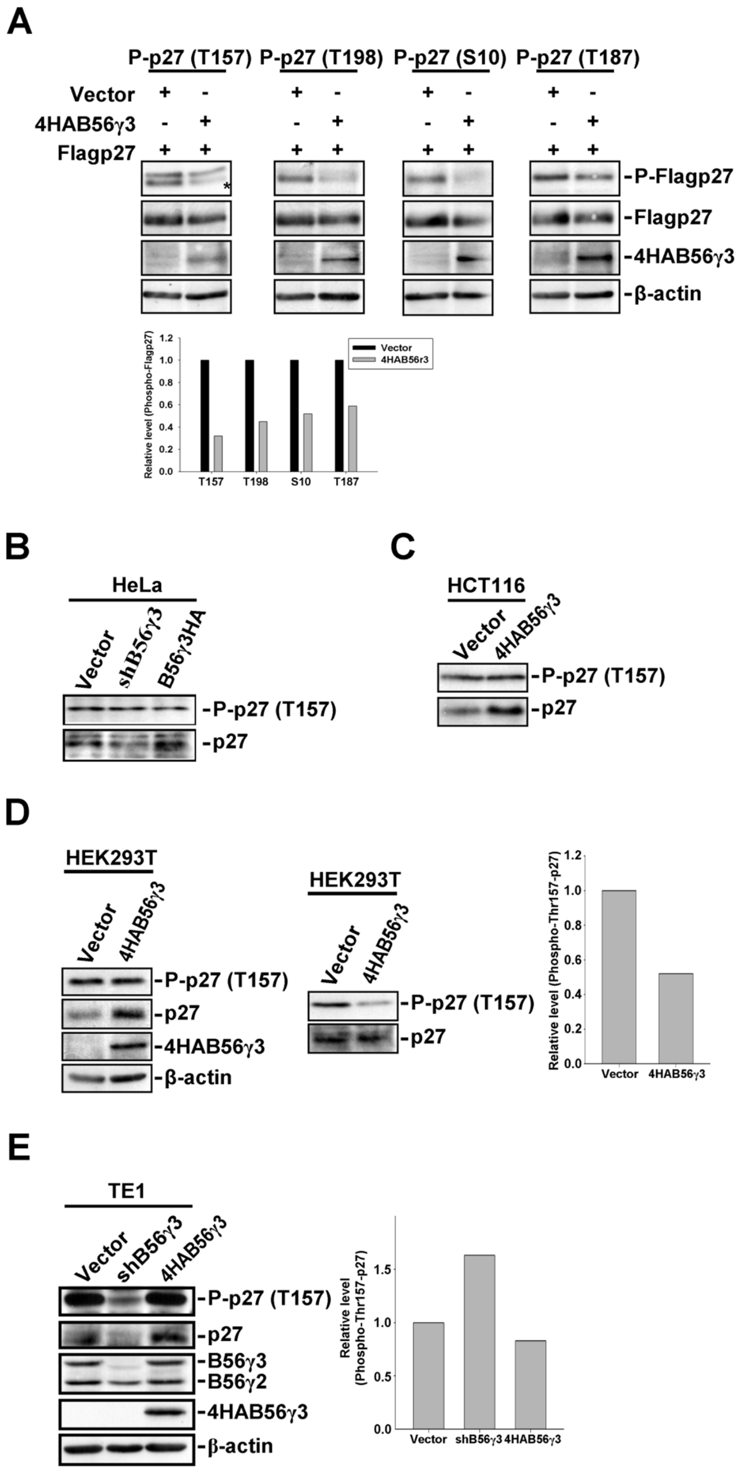

Supplementary Figure S2: PP2A-B56γ3 selectively regulates p27 phosphorylation at Thr157. A. Lysates of NIH3T3 cells transfected with expression vector of Flag-p27 and 4HA-B56γ3 or vector only were analyzed by SDS-PAGE and immunoblotting with antibodies specific for phospho-p27 (Thr187), phospho-p27 (Thr157), phospho-p27 (Thr198), phospho-p27 (Ser10), total p27, HA, B56γ, and β-actin. **B.** Lysates of HeLa cells with vector only, B56γ3 overexpression, or B56γ3 knockdown were analyzed for levels of phospho-p27 (Thr157), p27, HA, B56γ, and β-actin. **C–E.** Lysates of HCT116 (C), HEK293T (D), TE1 (E) cells with vector only, B56γ3 overexpression, or B56γ3 knockdown were analyzed as described above. The supplementary Figure B and C are data without adjustments for loading of Figure 2C and 2D of the article. The relative expression level of phospho-p27 were quantified by densitometry and normalized with total p27. The data shown are expressed as -fold expression level over that of vector control, which was set as 1. Data shown are from one representative experiment of at least two experiments with similar results.

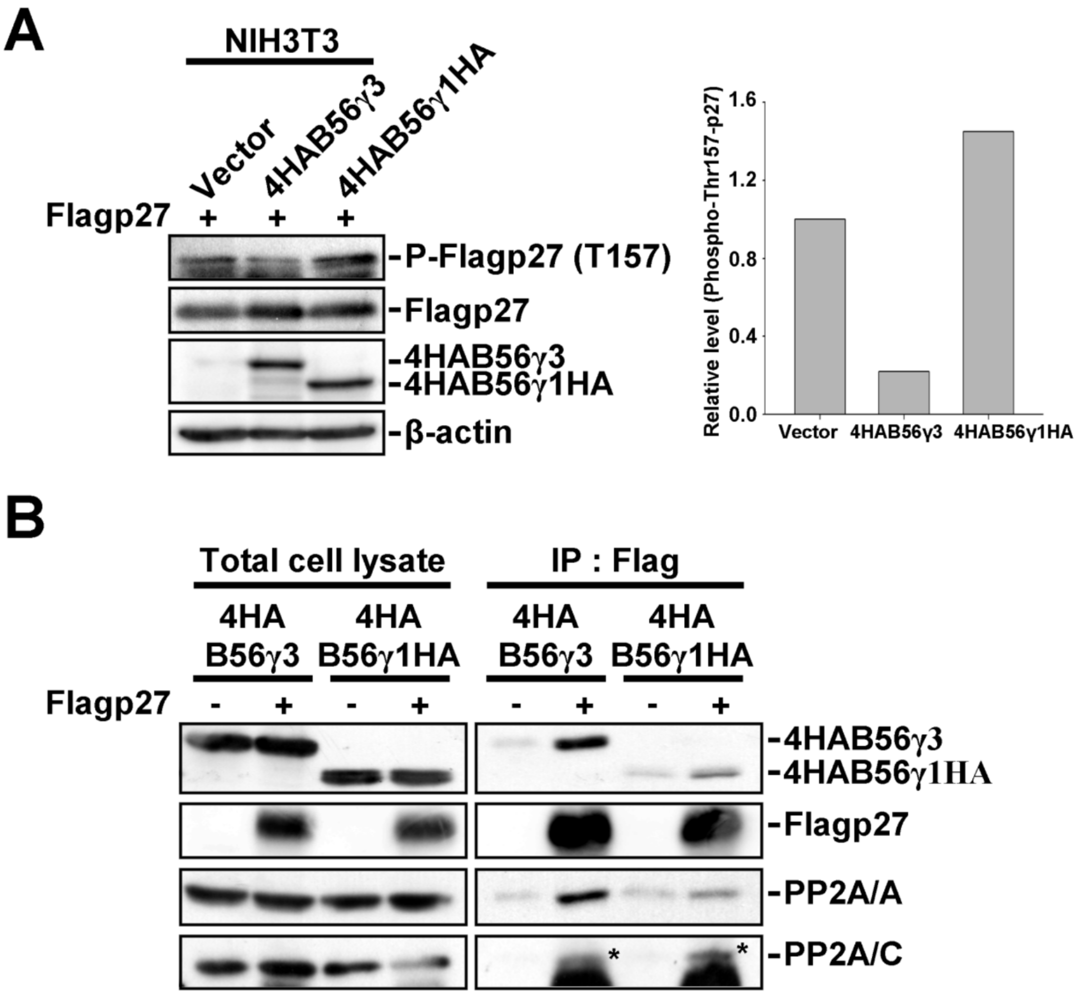

Supplementary Figure S3: Both B56γ3 and B56γ1 can interact with p27, but only B56γ3 dephosphorylates p27 at Thr157. A. NIH3T3 cells with vector only, B56γ3 overexpression, or B56γ1 overexpression transfected with expression vector of Flag-p27 or empty vector were analyzed by immunoblotting with antibodies specific for phospho-p27 (Thr157), p27, HA, and β-actin. The relative expression level of phospho-p27 was quantified by densitometry and normalized with total Flag-p27. The data shown are expressed as -fold expression level over that of vector control, which was set as 1. **B.** Lysates of NIH3T3 cells with B56γ3 overexpression or B56γ1 overexpression transfected with expression vector of Flag-p27 or empty vector were immunoprecipitated (IP) by anti-FLAG-Sepharose, and the immunocomplexes were analyzed by SDS-PAGE and immunoblotting by antibodies specific for HA, PP2A/Aα, FLAG, and PP2A/Cα. Data shown are from one representative experiment of at least two experiments with similar results.

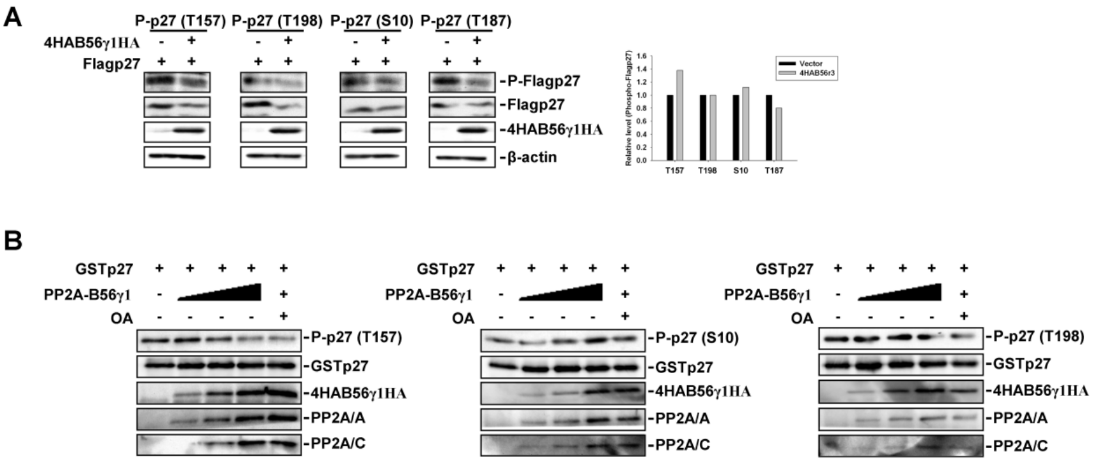

Supplementary Figure S4: B56γ1 regulates phosphorylation of p27 at Thr187, but not Thr157, Ser10, or Thr198. A. Lysates of NIH3T3 cells co-transfected with expression vectors of Flag-p27 and 4HA-B56γ1HA or empty vector were analyzed by immunoblotting with antibodies specific for phospho-p27 (Thr187), phospho-p27 (Thr157), phospho-p27 (Thr198), phospho-p27 (Ser10), p27, HA, and β-actin. The relative expression level of each phospho-p27 was quantified by densitometry and normalized with total Flag-p27 or endogenous p27. The data shown are expressed as -fold expression level over that of vector control, which was set as 1. **B.** *In vitro* dephosphorylation reactions, *in vitro* Akt phosphorylated GST-p27 proteins were incubated in the absence or presence of various amounts of B56γ1-containing PP2A complexes with or without 1 μM okadaic acid (OA) at 37 °C for 30 min. Expression levels of phospho-GSTp27 (Thr157), GST-p27, 4HA-B56γ1, and PP2A A and C subunits were detected by immunoblotting with antibodies specific for phospho-p27 (Thr157), phospho-p27 (Ser10), phospho-p27 (Thr198), GST, HA, PP2A/A and PP2A/C. The phosphorylation levels of p27 were quantified by densitometry and normalized with total p27. Levels of control reactions with no addition of PP2A-B56γ3 complexes were set as 100%. Data expressed as percentages of reduction of phospho-p27 in individual reactions in the presence of PP2A-B56γ1 complexes with or without OA. Data shown are from one representative experiment of at least two experiments with similar results.

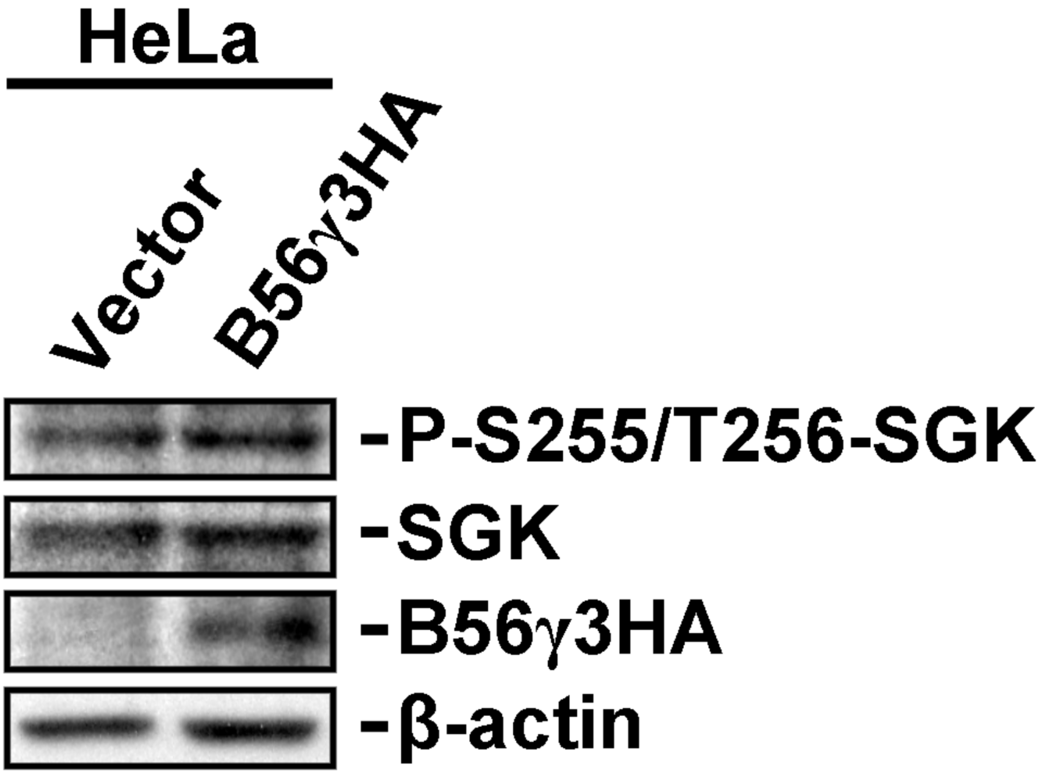

Supplementary Figure S5: B56γ3 does not regulate phosphorylation of SGK. Lysates of HeLa cells with vector only or B56γ3 overexpression were analyzed by immunoblotting with antibodies specific for phospho-SGK (Ser255/Thr256), SGK, HA and β-actin.
